# Developmental Changes of Glutamate and GABA Receptor Densities in Wistar Rats

**DOI:** 10.3389/fnana.2019.00100

**Published:** 2019-12-20

**Authors:** Sabrina Behuet, Jennifer Nadine Cremer, Markus Cremer, Nicola Palomero-Gallagher, Karl Zilles, Katrin Amunts

**Affiliations:** ^1^Institute of Neuroscience and Medicine (INM-1), Jülich Research Centre, Jülich, Germany; ^2^Cécile and Oskar Vogt Institute of Brain Research, Medical Faculty, Heinrich-Heine University, Düsseldorf, Germany

**Keywords:** receptor autoradiography, glutamate, GABA, neurotransmitter receptor, brain development, rat brain

## Abstract

Neurotransmitters and their receptors are key molecules of signal transduction and subject to various changes during pre- and postnatal development. Previous studies addressed ontogeny at the level of neurotransmitters and expression of neurotransmitter receptor subunits. However, developmental changes in receptor densities to this day are not well understood. Here, we analyzed developmental changes in excitatory glutamate and inhibitory γ-aminobutyric acid (GABA) receptors in adjacent sections of the rat brain by means of quantitative *in vitro* receptor autoradiography. Receptor densities of the ionotropic glutamatergic receptors α-amino-3-hydroxy-5-methyl-4-isoxazolepropionic acid (AMPA), kainate and N-methyl-D-aspartate (NMDA) as well as of the ionotropic GABA_A_ and metabotropic GABA_B_ receptors were investigated using specific high-affinity ligands. For each receptor binding site, significant density differences were demonstrated in the investigated regions of interest [olfactory bulb, striatum, hippocampus, and cerebellum] and developmental stages [postnatal day (P) 0, 10, 20, 30 and 90]. In particular, we showed that the glutamatergic and GABAergic receptor densities were already present between P0 and P10 in all regions of interest, which may indicate the early relevance of these receptors for brain development. A transient increase of glutamatergic receptor densities in the hippocampus was found, indicating their possible involvement in synaptic plasticity. We demonstrated a decline of NMDA receptor densities in the striatum and hippocampus from P30 to P90, which could be due to synapse elimination, a process that redefines neuronal networks in postnatal brains. Furthermore, the highest increase in GABA_A_ receptor densities from P10 to P20 coincides with the developmental shift from excitatory to inhibitory GABA transmission. Moreover, the increase from P10 to P20 in GABA_A_ receptor densities in the cerebellum corresponds to a point in time when functional GABAergic synapses are formed. Taken together, the present data reveal differential changes in glutamate and GABA receptor densities during postnatal rat brain development, which may contribute to their specific functions during ontogenesis, thus providing a deeper understanding of brain ontogenesis and receptor function.

## Introduction

Brain development is characterized by various molecular, cellular, structural as well as functional alterations underlying the acquisition of motor and cognitive skills during ontogenesis and increasingly complex interaction with the environment. Neurotransmitters and their receptors are key molecules of signal transduction. The neurotransmitters glutamate and γ-aminobutyric acid (GABA) are of particular interest in this context, due to the fact that they are key constituents of the balance between excitation and inhibition, and play a major role in different processes associated with brain development. For example, glutamate receptors are involved in neuronal migration and synaptogenesis (Luján et al., 2005) as well as in synaptic plasticity and thus in the modulation of memory and learning processes (Granger et al., 2013; Granger and Nicoll, 2014). GABA receptors are involved in controlling adult neurogenesis (Giachino et al., 2014), neuronal migration (López-Bendito et al., 2004; Gaiarsa and Porcher, 2013) as well as learning and memory (Heaney and Kinney, 2016). In addition to the individual roles of glutamate and GABA receptors, they are co-localized in many brain regions (Luján et al., 2005) and their balanced interaction is a key factor for normal brain development (Manent and Represa, 2007; Luhmann et al., 2015). Imbalances in glutamate and GABA impair brain function, potentially leading to brain pathology, for instance in epilepsy (Naylor, 2010) and post-traumatic stress disorder (Gao et al., 2014). Thus, the analysis of both neurotransmitter systems *via* a comprehensive set of different receptor types is necessary to get an insight into their complex interaction in mammalian brains.

According to their pharmacological agonist, there are three types of ionotropic glutamate receptors: α-amino-3-hydroxy-5-methyl-4-isoxazolepropionic acid (AMPA), kainate and N-methyl-D-aspartate (NMDA). AMPA receptors are cation-selective ionotropic receptors that mediate the majority of fast excitatory neurotransmission in the mammalian brain and are involved in synaptic plasticity (Huganir and Nicoll, 2013; Zhou et al., 2018), e.g., in motor learning processes in the cerebellum (Kano and Kato, 1987). Furthermore, they play a crucial role in excitatory synapse formation, stabilization and neuronal circuit formation due to changes in the quantity and nature of AMPA receptors (Henley and Wilkinson, 2016). AMPA receptors are located throughout the brain in the membranes of neurons and neuroglia, e.g., in oligodendrocytes and astrocytes (Petralia and Wenthold, 1992; Martin et al., 1993).

Kainate receptors are involved in hippocampal mossy fiber short- and long-term plasticity (Contractor et al., 2001) and expeditious changes in their trafficking might alter synaptic transmission during synaptic plasticity and neuronal development (Jane et al., 2009). In addition to their involvement in postsynaptic transmission, they contribute to the modulation of synaptic transmission and neuronal excitability (Jane et al., 2009; Contractor et al., 2011).

NMDA receptors have a high Ca^2+^ permeability, which is important for the regulation of synaptic plasticity (Lau et al., 2009). More precisely, the induction of long-term potentiation (LTP) at the CA1 synapses of the hippocampus (Muller et al., 1988; Zakharenko et al., 2001), or olfactory learning (Lincoln et al., 1988) is dependent on NMDA receptor activation. Furthermore, they are involved in experience-dependent changes, including cognition, neuronal differentiation and synapse consolidation in the developing brain (McDonald and Johnston, 1990; Planells-Cases et al., 2006). Interestingly, NMDA receptor densities and subunit expression levels change over the course of development (Laurie et al., 1997; Wenzel et al., 1997), thus making them a relevant target of ontogenetic studies.

The neurotransmitter GABA binds to and activates GABA receptors. GABA_A_ receptors mediate fast GABA responses, are primarily permeable to Cl^−^, and are involved in synaptic plasticity by changing the transmembrane Cl^−^ gradient and thus influence synaptic strength (Raimondo et al., 2012; Huang et al., 2013). During early brain development, GABA_A_ first acts as an excitatory neurotransmitter due to a higher intracellular than extracellular Cl^−^ concentration, and subsequently changes its action to inhibitory due to a reduction of intracellular Cl^−^ levels after the first postnatal week (Cherubini et al., 1991; Rivera et al., 1999). Interestingly, the first synapses to be formed and activated in the embryonic central nervous system are GABAergic (Khazipov et al., 2001). Moreover, the involvement of GABA_A_ receptors in cognitive processes such as memory formation or consolidation (Möhler, 2009), and a link between anxiety and memory (Kalueff and Nutt, 1996), as well as between GABA_A_ receptor subunit expression and water maze performance as a task for spatial learning (Collinson et al., 2002) were indicated. Hence, knowledge about alterations in GABA_A_ receptor densities during brain development may contribute to a better understanding of their specific role in ontogenesis. GABA_B_ receptors regulate Ca^2+^ and/or K^+^ channel conductance in the membrane and can inhibit the release of neurotransmitters and thus neuronal activity (Ulrich and Bettler, 2007). Their inhibitory effect is slower and longer-lasting compared to GABA_A_ inhibition (Simeone et al., 2003), and found both at pre- and postsynaptic sites in the hippocampus (López-Bendito et al., 2004). They are involved in brain development by controlling adult neurogenesis (Giachino et al., 2014) and neuronal migration (López-Bendito et al., 2004; Gaiarsa and Porcher, 2013). Furthermore, GABA_B_ receptors have also been implicated in learning and memory processes, as reviewed by Heaney and Kinney (2016). For instance, GABA_B1a_ and GABA_B1b_ receptor knockouts were impaired in the performance of a working memory task, indicating that the GABA_B1_ receptor subtypes are essential for correct task performance (Jacobson et al., 2007; Heaney and Kinney, 2016). The analysis of neurotransmitter levels, in general, indicated that glutamate and GABA are abundant and widespread in both the pre- and postnatal brain (Miranda-Contreras et al., 1999; Miranda-Contreras et al., 2000).

However, despite previous studies focusing on ontogenetic alterations in receptor functions, subunit constellations and binding properties, our understanding is still incomplete, e.g., with respect to regional density differences and time intervals of varying expression levels (Insel et al., 1990; Pellegrini-Giampietro et al., 1991; Miranda-Contreras et al., 2000). An increase in striatal AMPA receptor density was revealed in caudate-putamen from P1 to P7, followed by a slight increase that peaked at P28 (Insel et al., 1990). In addition, differential changes in hippocampal subregions were revealed, as the densities in CA1 and DG peaked at P28, whereas the densities in CA3 peaked at P14, with a subsequent decrease until P60 (Insel et al., 1990). Moreover, an association between LTP and increased AMPA receptor densities in several hippocampal subregions and cortical areas was observed in rats that exhibited LTP after receiving high-frequency theta burst stimulation to the perforant pathway (Tocco et al., 1992). Xia and Haddad (1992) demonstrated that GABA_A_ receptor densities in the cerebellum and rostral brain areas such as the neocortex, thalamus, and dentate gyrus increased with age, particularly between P10 and P21, whereas no significant increases were found in the striatum and hippocampal CA1 region (Xia and Haddad, 1992). GABA_B_ receptor densities of the striatum and hippocampus peaked at P7 and decreased significantly from P28 to adults. Taken together, these findings suggest a complex pattern of regionally specific changes during ontogeny.

Therefore, the present study addresses ontogenetic alterations of different receptor types for glutamate and GABA by using quantitative *in vitro* receptor autoradiography to characterize their regional patterns of developmental changes in more detail. For this purpose, neighboring sections of the same rat brains were used to analyze receptors of both glutamate and GABA, thus enabling for the first time direct comparison while eliminating inter-subject and methodical differences. In contrast to earlier ontogenetic studies that mostly used non-specific or low-affinity [^3^H]ligands, the present study focused on specific [^3^H]ligands with high affinities to their respective neurotransmitter receptors. We investigated their densities in the following regions of interest (ROIs): olfactory bulb (OB), striatum (caudate-putamen, CPu), hippocampus (Hip) and cerebellum (Cb) at multiple developmental stages (postnatal day (P) 0, 10, 20, 30 and 90). The color-coded images in combination with the densitometric analysis of the autoradiograms enabled a specific anatomical mapping of receptor density alterations in different brain regions.

## Materials and Methods

### Animals

All experiments were performed according to the German animal welfare act and were approved by the responsible governmental agency, LANUV NRW (Regional authorities for nature, environment and consumer protection NRW, Germany). All animals were kept under standard laboratory conditions with access to food and water *ad libitum*.

In the present study, we used a total of 25 Wistar rats (Charles-River, Germany), with five animals being assigned to each of the following age groups: postnatal day 0 (P0; 10 ± 1 g body weight), P10 (24 ± 2 g body weight), P20 (50 ± 3 g body weight), P30 (105 ± 6 g body weight) and P90 (250 ± 10 g body weight). The P0 and P10 rats were decapitated without sedation, according to the German animal welfare act. The P20, P30 and P90 rats were sedated by carbon dioxide (CO_2_) inhalation and subsequently decapitated. The P0 heads were immediately deep-frozen in −50°C isopentane, and stored at −80°C. The P10, P20, P30 and P90 brains were immediately removed from the skull, hemispheres were separated, deep-frozen in −50°C isopentane, and stored at −80°C. Only the left hemisphere was used in the present study.

**Table 1:** Receptor binding protocols for glutamatergic and GABAergic [^3^H]ligands, including displacer (marked with *) and incubation conditions.

Receptor-[^3^H]ligand	Procedure	Incubation buffer	Time/Temperature
	Fixation	4% neutral buffered formalin	30 min at 22°C
	Pre-incubation	50 mM Tris-acetate (pH 7.2)	3 × 10 min at 4°C
	Main incubation	50 mM Tris-acetate (pH 7.2)	45 min at 4°C
		+ 100 mM KSCN	
AMPA-[^3^H]AMPA		+ 10 nM [^3^H]AMPA	
		+ 10 μM (+)Quisqualate*	
	1st Rinsing	50 mM Tris-acetate (pH 7.2)	4 × 4 s at 4°C
	2nd Rinsing	2.5% Glutaraldehyde in acetone	2 × 2 s at 22°C
	Fixation	4% neutral buffered formalin	30 min at 22°C
	Pre-incubation	50 mM Tris-citrate (pH 7.1)	3 × 10 min at 4°C
	Main incubation	50 mM Tris-citrate (pH 7.1)	45 min at 4°C
		+ 10 mM Calcium acetate	
Kainate-[^3^H]Kainic acid		+ 9.4 nM [^3^H]Kainic acid	
		+ 100 μM SYM 2081*	
	1st Rinsing	50 mM Tris-citrate (pH 7.1)	3 × 4 s at 4°C
	2nd Rinsing	2.5% Glutaraldehyde in acetone	2 × 2 s at 22°C
	Pre-incubation	50 mM Tris-HCl (pH 7.2)	15 min at 4°C
		+ 50 μM Glutamate	
	Main incubation	50 mM Tris-HCl (pH 7.2)	60 min at 22°C
		+ 50 μM Glutamate	
		+ 30 μM Glycine	
NMDA-[^3^H]MK-801(+)		+ 50 μM Spermidine	
		+ 3.3 nM [^3^H]MK-801(+)	
		+ 100 μM (+)MK-801*	
	1st Rinsing	50 mM Tris-HCl (pH 7.2)	2 × 5 min at 4°C
		+ 50 μM Glutamate	
	2nd Rinsing	Distilled water	1 × dip at 4°C
	Fixation	4% neutral buffered formalin	30 min at 22°C
	Pre-incubation	50 mM Tris-citrate (pH 7.0)	3 × 5 min at 4°C
	Main incubation	50 mM Tris-citrate (pH 7.0)	40 min at 4°C
GABA_A_-[^3^H]SR 95531		+ 3 nM [^3^H]SR 95531	
		+ 1 mM GABA*	
	1st Rinsing	50 mM Tris-citrate (pH 7.0)	3 × 3 s at 4°C
	2nd Rinsing	Distilled water	1 × dip at 4°C
	Pre-incubation	50 mM Tris-HCl (pH 7.2)	3 × 5 min at 4°C
		+ 2.5 mM CaCl_2_	
	Main incubation	50 mM Tris-HCl (pH 7.2)	60 min at 4°C
		+ 2.5 mM CaCl_2_	
GABA_B_-[^3^H]CGP 54626		+ 2 nM [^3^H]CGP 54626	
		+ 100 μM CGP 55845*	
	1st Rinsing	50 mM Tris-HCl (pH 7.2)	3 × 2 s at 4°C
		+ 2.5 mM CaCl_2_	
	2nd Rinsing	Distilled water	1 × dip at 4°C

### Tissue Processing

The tissue was serially sectioned (20 μm section thickness) at −13°C in the coronal plane using a cryostat microtome (Leica, Germany). For each animal and ROI, we prepared six sections for autoradiographic experiments (five sections for the total binding and one section for the unspecific binding, see below) and five sections for the histological staining. Each section was mounted on a pre-cooled, silanized glass slide and dried on a heating plate at 37°C for 30 min. The sections were kept in vacuum-sealed plastic bags in a freezer at −80°C until their use for either histological cresyl violet staining or quantitative *in vitro* receptor autoradiography (Zilles et al., 2002; Palomero-Gallagher et al., 2003; Palomero-Gallagher and Zilles, 2018).

### Quantitative *in vitro* Receptor Autoradiography

The receptor binding sites of the glutamatergic AMPA, kainate and NMDA receptors as well as the GABAergic GABA_A_ and GABA_B_ receptors were labeled by using previously described protocols (Zilles et al., 2002, 2004; Palomero-Gallagher and Zilles, 2018). The respective tritiated ligands [^3^H]AMPA, [^3^H]Kainic acid, [^3^H]MK-801(+) and [^3^H]SR 95531 were purchased from PerkinElmer (Germany), and [^3^H]CGP 54626 was acquired from Biotrend (Germany).

The receptor autoradiographic method consists of three steps. During the pre-incubation, the sections were rehydrated and the endogenous ligands were washed out. In the main incubation, two different protocols were used to determine specific binding: one for the total binding, where the sections were labeled solely with the tritiated receptor ligand, and one for the unspecific binding, where a specific unlabeled displacer was also added. Finally, sections underwent a rinsing step in buffer to stop the binding process by removing excess tritium labeled ligand. For visualization of NMDA, GABA_A_ and GABA_B_ receptors, this step was followed by a rinsing in distilled water to wash out buffer salts. In the case of AMPA and kainate receptor, the rinsing step in buffer was followed by a fixation in 2.5% glutaraldehyde/acetone. Details of the binding protocols are summarized in Table 1.

The following regions of interest were selected due to their respective roles in learning and memory processes: olfactory bulb, striatum, hippocampus, and cerebellum. Previous studies demonstrated a link between the olfactory bulb, memory, and emotion (Jaako-Movits and Zharkovsky, 2005). Amongst others, the striatum is important in the assistance of body movement and potentially involved in working memory (Packard and Knowlton, 2002). The hippocampus plays an important role in the formation of short-term and long-term memory as well as spatial navigation (Henke et al., 1999), while the cerebellum is involved in controlling motor functions and in motor learning, also known as implicit learning (Timmann et al., 2010).

### Histology

In order to localize and analyze the regions of interest *via* light microscopy as well as in the autoradiographs, Nissl staining with cresyl violet was carried out. After the frozen rat brain sections were acclimatized to room temperature, they were fixed in 4% neutral buffered formalin for 30 min. Subsequently, sections were washed in distilled water for 15 min and then incubated in a 0.1% cresyl violet staining solution for at least 5 min. After rinsing in distilled water for a few seconds, they were differentiated in 70% isopropyl alcohol. The sections were subsequently dehydrated in increasing isopropyl alcohol concentrations (80%, 90%, 96%, 100%), each for 1 min. Finally, sections were placed for 10 min in the intermedium XEM (surrogated for xylene) and then coverslipped with DPX (Distrene, Plasticizer, Xylene). The Nissl stained sections were digitized with a color camera for high-resolution images (~5 μm) that were taken with a sensitive CCD-sensor (Carl Zeiss Microscopy GmbH, Germany).

### Image Acquisition and Data Analysis

Tritium labeled sections were co-exposed with microscales of known radioactive concentrations against imaging plates (Fujifilm Corporation, Japan) for 72 h. After the exposition, the imaging plates were scanned with the BAS 5000 Bio-Imaging Analyzer System (Fujifilm Corporation, Japan).

AIDA Image Analyzer v.4.13 software (Elysia-raytest GmbH, Germany) was used for densitometric analysis of the ensuing autoradiograms. The gray values of the background of the imaging plates and the microscales (in ascending order of concentration) were measured in AIDA Image Analyzer v.4.13 software to compute linear regression curves. These are necessary to convert the intensity per area (PSL/Pixel) within each ROI into corresponding concentrations of radioactivity and finally into receptor densities (fmol/mg protein). Receptor densities were then averaged for five sections per animal, receptor type and ROI, and mean values were thus obtained for each receptor subtype.

**Figure 1:**
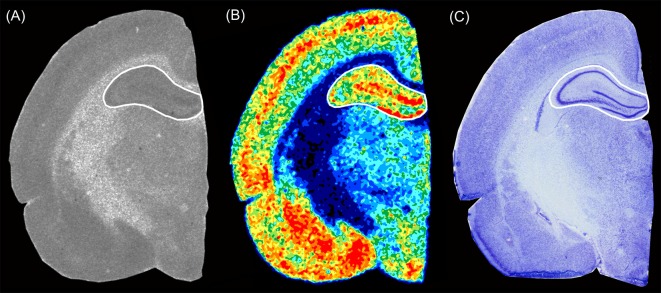
Exemplary section from a P20 rat brain showing the hippocampal level of **(A)** an autoradiographic image of [^3^H]SR 95531 binding to GABA_A_ receptors, **(B)** the respective color-coded image of the same autoradiographic image, and **(C)** an adjacent Nissl stained section. The Paxinos and Watson rat brain atlas (Paxinos and Watson, 2005) was used to verify the regions of interest.

To analyze the ROIs, we compared the original autoradiogram with its color-coded image, the neighboring Nissl stained section and the Paxinos and Watson rat brain atlas (Paxinos and Watson, 2005) to ensure that we respected the boundaries to adjacent brain regions (Figure 1). The olfactory bulb, the hippocampus, and the cerebellum were analyzed entirely without considering subregions or layers, since their boundaries were not easily recognizable given the limited resolution of the imaging plates, especially in the brains of P0 and P10 animals. Therefore, a quantitative analysis may have been error-prone even if differences seemed to be observed visually. To give an example: the color-coded autoradiogram of GABA_A_ receptors densities in a P20 rat brain revealed that the molecular layer of the dentate gyrus had a higher receptor density than the pyramidal layer of the CA1 and CA2/3 regions (Figure 1). However, it was not possible to recognize these boundaries in P0 and P10 brains as these regions only occupied a few pixels.

Significant differences between postnatal stages were analyzed for each receptor type separately by using an analysis of variance (ANOVA) with randomized block design as an omnibus test in which the stages of development were set as a between factor and the analyzed brain regions as a within factor. In the case of a significant result, the ROIs were analyzed individually for each receptor type. In the case of a significant result, a second ANOVA (without defined factors) was followed by *post hoc* *t*-tests to identify regions showing significant developmental changes. Threshold was set at *p* < 0.05, and the results of the ANOVA tests were Bonferroni corrected.

To illustrate the regional variations of receptor densities in different stages of development, autoradiograms were contrast-enhanced and color-coded using MATLAB^®^-software (MathWorks, Germany). A color bar, which is subdivided into 11 equally spaced density ranges, encodes the receptor density in fmol/mg protein.

## Results

**Figure 2:**
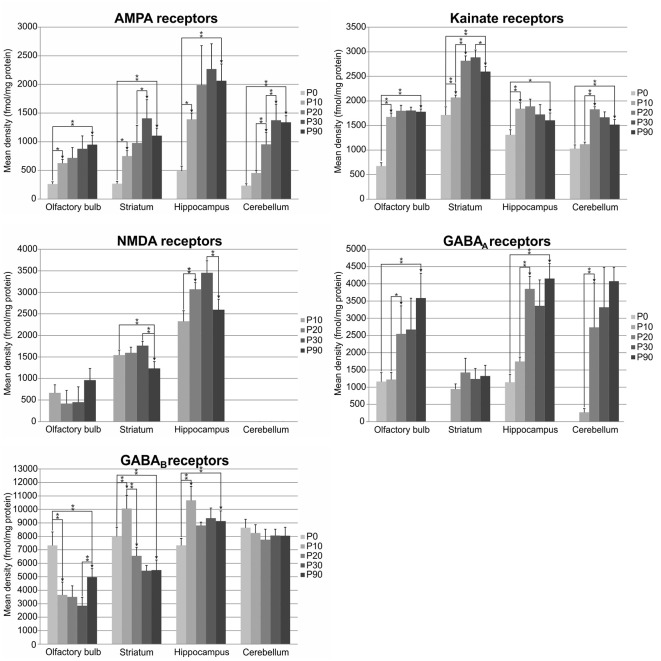
Bar charts depicting mean (and standard deviation) glutamatergic and GABAergic receptor densities in fmol/mg protein at different developmental stages (postnatal day of Wistar rat: P0, P10, P20, P30 and P90; each age group was represented with *n* = 5) and different brain regions (olfactory bulb, striatum, hippocampus and cerebellum). Significant differences are indicated by asterisks (*p < 0.05 or **p < 0.01).

**Figure 3:**
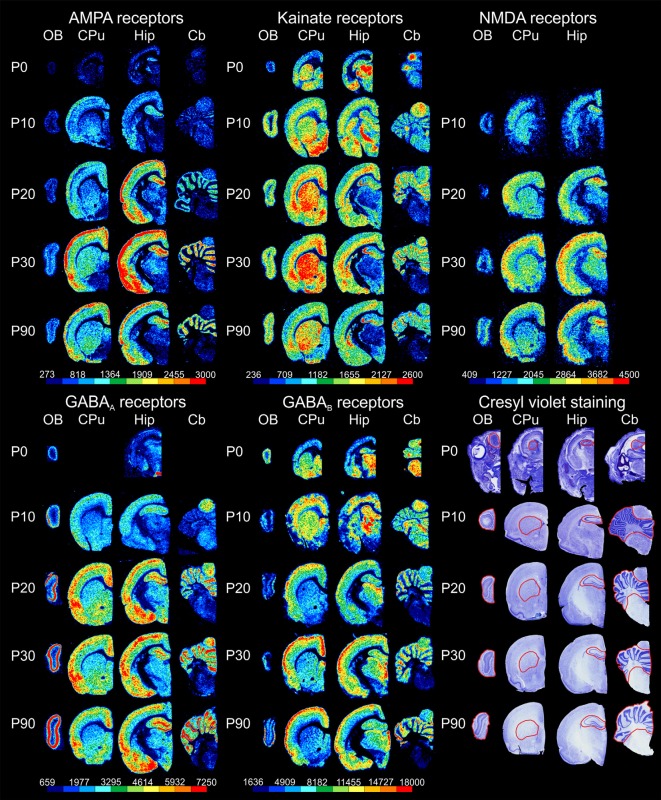
Exemplary sections through representative rostrocaudal levels for the examined brain regions [olfactory bulb (OB), striatum (caudate-putamen, CPu), hippocampus (Hip) and cerebellum (Cb)] depicting color-coded autoradiographic images of glutamatergic and GABAergic receptor densities as well as images of cresyl violet staining at different developmental stages (postnatal day of Wistar rat: P0, P10, P20, P30 and P90). Scale bars code for receptor densities in fmol/mg protein.

Receptor autoradiography and subsequent densitometric analysis revealed alterations in the densities of receptors for glutamate and GABA during postnatal brain development. For almost all ROIs, significant alterations were found for every receptor type analyzed. The densities of most receptors peaked within the first 30 postnatal days, except for GABA_A_ receptor densities, which reached the highest values at P90. The bar charts in Figure 2 show the receptor densities (mean and standard deviation given in fmol/mg protein; each age group was represented with *n* = 5) of the glutamatergic AMPA, kainate and NMDA receptors, as well as of the GABAergic GABA_A_ and GABA_B_ receptors. Significant differences between contiguous age groups (e.g., comparison of P0 and P10, then P10 and P20, etc.) and between P0 and P90 are indicated. Exemplary color-coded autoradiographs from representative rostrocaudal levels for each area and receptor type are depicted in Figure 3.

AMPA receptor densities were significantly higher (*p* < 0.01) in P90 rats compared to P0 in all brain regions investigated (olfactory bulb: 262%; striatum: 311%; hippocampus: 321% and cerebellum: 471%). Although the course of changes between age groups was comparable in all examined areas, their peak varied for the different brain regions (Figures 2, 3). Between P0 and P10, a significant increase was found in the olfactory bulb (139%, *p* < 0.05), the striatum (179%, *p* < 0.05) and the hippocampus (184%, *p* < 0.05), but not in the cerebellum. Significant changes were found between P10 and P20 only in the cerebellum (110% increase, *p* < 0.01) and between P20 and P30 in the striatum (44%, *p* < 0.05) and cerebellum (44%, *p* < 0.01; Figures 2, 3). Finally, between P30 and P90, AMPA receptor densities decreased in the striatum, hippocampus and cerebellum, though changes did not reach the level of significance.

Kainate receptor densities were significantly higher in P90 rats compared to P0 in all brain regions investigated (olfactory bulb: 164%, *p* < 0.01; striatum: 51%, *p* < 0.01; hippocampus: 22%, *p* < 0.05; cerebellum: 47%, *p* < 0.01). As described for the AMPA receptors, the course of changes between age groups was comparable in all examined areas, but their peaks varied for the different brain regions (Figures 2, 3). Between P0 and P10, densities increased significantly in the olfactory bulb (148%, *p* < 0.01), striatum (21%, *p* < 0.01) and hippocampus (41%, *p* < 0.01). Furthermore, between P10 and P20, a significant increase was found only in the striatum (36%, *p* < 0.01) and cerebellum (63%, *p* < 0.01), whereas no significant changes were found between P20 and P30. Interestingly, between P30 and P90, there was a decrease in receptor densities, though it only reached significance in the striatum (−10%, *p* < 0.05; Figures 2, 3).

NMDA receptor densities were below the detection limit in the brain of P0 rats and in the cerebellum at all ages. Interestingly, time-courses of changes in the striatum and hippocampus differed from those detected in the olfactory bulb (Figures 2, 3). In the olfactory bulb, NMDA receptor densities were higher at P90 than P10, whereas the opposite held true for the striatum and hippocampus. However, only the changes in the striatum reached significance (−23%, *p* < 0.01). Between P10 and P20, NMDA receptor densities increased significantly in the hippocampus (32%, *p* < 0.01). No significant changes were found between P20 and P30. Between P30 and P90, a significant decrease was found in the striatum (−30%, *p* < 0.01) and hippocampus (−25%, *p* < 0.01; Figures 2, 3).

GABA_A_ receptor densities were below the detection limit in the striatum and cerebellum of P0 rats. The course, but not the peak, of changes between age groups, was comparable in all areas examined (Figures 2, 3). GABA_A_ receptor densities were significantly higher in P90 rats compared to P0 in the olfactory bulb (208%, *p* < 0.01) and the hippocampus (263%, *p* < 0.01). No significant changes were found between P0 and P10, between P20 and P30, or between P30 and P90. Finally, between P10 and P20, a significant increase was detected in the olfactory bulb (108%, *p* < 0.05), hippocampus (121%, *p* < 0.01) and cerebellum (907%, *p* < 0.01; Figures 2, 3).

GABA_B_ receptor changes presented different time-courses and peaks in the examined regions (Figures 2, 3). In the olfactory bulb and striatum GABA_B_ receptor densities were significantly lower in P90 rats compared to P0 (olfactory bulb: −32%, *p* < 0.01; striatum: −31%, *p* < 0.01), whereas the opposite held true for the hippocampus (25%, *p* < 0.01). Interestingly, between P0 and P10, GABA_B_ receptor densities decreased significantly in the olfactory bulb (−50%, *p* < 0.01), but increased significantly in the striatum (26%, *p* < 0.01) and hippocampus (46%, *p* < 0.01). Between P10 and P20, GABA_B_ receptor densities decreased significantly in the striatum (−35%, *p* < 0.01). No significant changes were found between P20 and P30. Between P30 and P90, there was a significant increase of GABA_B_ receptor densities in the olfactory bulb (74%, *p* < 0.01; Figures 2, 3).

## Discussion

The present study investigated ontogenetic alterations of five receptor types of the two major neurotransmitter systems, glutamate and GABA, in different regions of Wistar rat brains by using quantitative *in vitro* receptor autoradiography. For the first time, ontogenetic changes in the densities of different receptor types of these two major neurotransmitters were comprehensively analyzed in adjacent sections using specific high-affinity tritiated ligands that allowed simultaneous analysis, while methodical and inter-subject alterations were avoided. The results indicated different developmental patterns and regional differences in receptor densities with numerous significant alterations for all investigated receptors.

AMPA receptor densities were significantly lower in newborns compared to adult rats. This is in accordance with the results of an earlier study, although the authors labeled the low-affinity binding site of the AMPA receptor (Insel et al., 1990), whereas the present study examined the high-affinity binding site. A developmental increase in hippocampal receptor densities could be explained by high levels of high affinity [^3^H]AMPA binding sites predominantly located in hippocampal cell body layers, reflecting changes in the relative numbers of binding sites rather than developmental changes in affinities for AMPA receptors (Standley et al., 1995).

Previous studies investigated the developmental alterations in the AMPA receptor subunits and revealed that their expression is developmentally regulated and varies depending on the brain region (Pellegrini-Giampietro et al., 1991; Martin et al., 1998; Pickard et al., 2000; Ritter et al., 2002). For instance, both the increased hippocampal gene expressions for GluA1 and GluA2 at P14 and for GluA3 at P21 (Pellegrini-Giampietro et al., 1991), as well as the alterations in receptor densities we found in the present study correlate with known periods of plasticity (Pellegrini-Giampietro et al., 1991). At the beginning of synaptic plasticity, NMDA receptors are activated through the depolarization of the postsynaptic membrane that is mediated by AMPA receptors, which then triggers a Ca^2+^-dependent signal pathway and leads to alterations of the postsynaptic membrane surface of AMPA receptors (Rao and Finkbeiner, 2007). Consequently, the postsynaptic surface modification of AMPA receptors leads to alterations in LTP and long-term depression (LTD), thus in synaptic strength (Rao and Finkbeiner, 2007). Numerous AMPA receptors in immature brains are permeable to Ca^2+^ due to their lacking the GluA2 subunit (Pellegrini-Giampietro et al., 1992). Furthermore, P10–P12 rats showed a significantly lower GluA2 expression in neocortex and hippocampus compared to adults (Sanchez et al., 2001) and after the 2nd postnatal week, there is an exchange of GluA2 lacking AMPA receptors with GluA2 containing (Ca^2+^ impermeable) receptors (Pellegrini-Giampietro et al., 1992). Hence, this switch may indicate the importance of AMPA receptors in the early postnatal brain development and especially in processes like synaptogenesis (Durand and Zukin, 1993; Simeone et al., 2004), and in the regulation of synaptic plasticity (Planells-Cases et al., 2006). Synaptogenesis is a complex process in which new synapses are formed, predominately during pre- and early postnatal brain development, but can also occur in mature brains, where it is important for learning, memory, and cognition (Waites et al., 2005). Taken together with the results of the present study, this developmental switch may correlate with the significant increase in receptor densities between P0 and P10, especially in the striatum and the hippocampus.

The developmental alterations in cerebellar AMPA receptor densities found in the present study are in accordance with the expression levels of genes coding for the GluA1–3 subunits since in both cases a continuous increase occurs between P0 and P30, followed by a slight but non-significant decrease to adult levels (Durand and Zukin, 1993). It is well known that the cerebellum plays a pivotal role in processes such as controlling motor functions, motor learning and movement (Matsumura et al., 2004; Timmann et al., 2010). We assume that the age-related AMPA receptor density increase in the cerebellum could be related to these functions due to its known role in cerebellar plasticity, in particular through its involvement in motor learning (Kano and Kato, 1987; Hirano, 2013).

The pattern of kainate receptor density changes was comparable in the hippocampus and striatum, showing a gradual increase until reaching a maximum at P20 and P30, respectively, followed by a decrease during adulthood, although the decrease was only significant from P30 to P90 in the striatum. These findings correlate with those of an ontogenetic study, where a similar pattern was found in the hippocampus and striatum with a peak at P21, potentially being correlated with cytoarchitectural reorganization occurring in these areas, and followed by a decline until adulthood (Miller et al., 1990). Furthermore, the time pattern with increased kainate receptor densities from P0 to P10 and P20 corresponds to periods of synaptogenesis (Fiala et al., 1998) which is also enhanced by LTP in the hippocampus of P15 rats (Watson et al., 2016). Interestingly, the significant increase of kainate receptor densities in the olfactory bulb described here, follows a similar time-course to that of the changes in synaptic density in the glomerulus (Moriizumi et al., 1995).

Ontogenetic changes in the expression patterns of the kainate genes (GluK1–5) and of kainate receptor densities have been studied in the past (Bahn et al., 1994). Regarding the latter, our results revealed a similar time-course in the hippocampus as they peaked in the early stages and then decreased until adult levels were reached. The transient changes in expression levels of kainate binding sites suggest the importance of kainate during neuronal development, and may indicate involvement in postnatal plasticity (Carta et al., 2014). Kainate receptors are able to regulate synaptic inhibition by controlling the expression of the K^+^-Cl^−^ cotransporter KCC2 (Rivera et al., 1999) and to mediate LTP in the hippocampal mossy fiber synapses (Bortolotto et al., 1999). Compared to AMPA and NMDA receptors, kainate receptors are less well studied and many questions remain unanswered. The present study may indicate their importance in brain development and could, therefore, provide reference data for future studies.

The significant changes in NMDA receptor densities in the striatum and hippocampus are proposed as alterations of receptor numbers (Insel et al., 1990; Colwell et al., 1998), as the possibility of developmental changes in ligand-receptor affinities expressed as dissociation constant was excluded (Tremblay et al., 1988). The non-specific glutamate and NMDA ligand L-[^3^H]glutamate was frequently used in earlier ontogenetic studies regarding NMDA receptors. However, by using the highly receptor-subtype specific ligand [^3^H]MK-801(+), a non-competitive antagonist that binds to the phencyclidine binding sites within the NMDA receptor ion channel, false-positive results are excluded. Moreover, NMDA receptors are unique in that they are not activated unless their co-agonists glutamate and glycine bind to their recognition site (Qü, 1995; Danysz and Parsons, 1998). In contrast to earlier ontogenetic studies, we have therefore included both co-agonists as well as the enhancer spermidine in our binding protocol (Qü, 1995). Baudry et al. (1981) proposed that NMDA receptor binding sites in the hippocampus are low in P4 rats, increase rapidly until P10, and then increase more slowly for the next 2–3 weeks. This is in line with our results showing NMDA receptor levels below the detection limit in all ROIs at P0. Electrophysiological studies strengthen this hypothesis since there was no response from most cells of the striatum to synaptic stimulation in rats before P10 (Hurst et al., 2001). Moreover, Maragos et al. (1988) investigated the regional distribution of two different NMDA receptor ligands and showed that the cerebellum was the only region in which NMDA binding sites did not coincide. By using the non-specific ligand L-[^3^H]glutamate, NMDA binding sites were measured in the cerebellar granule cell layer, whereas only a few binding sites were measured with [^3^H]TCP, a ligand that binds to the phencyclidine binding site of NMDA receptors (Maragos et al., 1988). Another study indicated that by using 5 nM of the NMDA specific ligand [^3^H]MK-801(+), which is comparable to the 3.3 nM in the present study, the granule cell layer of the cerebellum was absent (Sakurai et al., 1991). However, by increasing the ligand concentration to 20 nM, they observed labeled cerebellar granule cell layers, indicating a low-affinity binding site in the cerebellum and thus a different receptor subtype composition compared to other brain regions (Sakurai et al., 1991). Indeed, it was demonstrated that besides a lower expression of NR2A, the cerebellum expresses mostly NR1-NR2C subunits that are, however, barely expressed in the cerebrum (Laurie et al., 1997; Paoletti et al., 2013). Furthermore, it was demonstrated that the NMDA receptor channel blocker [^3^H]MK-801(+) exhibits high affinities for recombinant heteromeric NR1-NR2A and NR1-NR2B receptors that are mainly expressed in the cerebrum and over 25-fold lower affinities for NR1-NR2C receptors that are mainly expressed in the cerebellum. These results indicate the heterogeneity of NMDA receptors with regard to different ligand affinities in different brain regions (Laurie and Seeburg, 1994). The differential subunit composition of NMDA receptors in different brain regions during brain development could be addressed in future studies, e.g., by using *in situ* hybridization or immunohistochemistry. In summary, we assume that NMDA receptor densities were below the detection limit in the cerebellum of both postnatal and adult rats for mainly two reasons. First, the fact that the highly specific NMDA receptor ligand [^3^H]MK-801(+) was used instead of the non-specific ligand L-[^3^H]glutamate in order to avoid false-positive results. Second, due to the probably different subunit composition and the resulting varying affinities in the cerebellum compared to the other brain regions analyzed.

The analysis of mRNA expression levels of NMDA receptor subunits revealed NR2B expression predominately in the first postnatal week, and NR2A expression in the following weeks (Wenzel et al., 1997; Ritter et al., 2002; Gambrill and Barria, 2011). This subunit switch is thought to play a developmental role in NMDA neurotoxicity (Haberny et al., 2002) and indicates the importance of neonatal neurotransmission mediated by the NMDA receptor (Wenzel et al., 1997). NR2A and NR2B subunit expression peaks in the 2nd postnatal week (Ritter et al., 2002), which may correlate with the time frame during which hippocampal synaptic plasticity is increased (Harris and Teyler, 1984). This idea is supported by a study of NR2A knockout mice, which showed a decrease in hippocampal LTP and spatial learning (Sakimura et al., 1995). As rats start to explore their surroundings between P15 and P28, synaptic density increases significantly in the hippocampus as a result of NMDA receptor activation (Steward and Falk, 1991). These developmental changes correlate with synaptogenesis (Gambrill and Barria, 2011), and could also be reflected by the increase in receptor densities which we found in the present study between P10 and P30. Like synaptogenesis, synapse elimination is another key process in normal brain development, as shown in a study addressing the number of synapses in the brain (Waites et al., 2005). During synaptogenesis, there is an increase in the numbers of synapses until reaching its peak after P28, but the numbers then decline slowly during maturation, a process known as synapse elimination (Steward and Falk, 1991; Lohmann and Kessels, 2014). Hence, the decline of receptor densities in striatum and hippocampus from P30 to P90 may be due to synapse elimination to redefine or rather fine-tune neuronal networks. Moreover, NMDA receptor activation results in the insertion of AMPA receptors and alters dendritic spine morphology, which is important for the stability and maturation of synapses (Waites et al., 2005).

We found the most significant increase in GABA_A_ receptor densities to take place between P10 and P20. By using the example of the hippocampus, it is known that during the first two postnatal weeks, synapses increase steadily in number (Steward and Falk, 1991; Fiala et al., 1998). Interestingly, this also coincides with the period in which the activation of this receptor type changes from being excitatory to inhibitory in nature, indicating that this shift plays an important role in brain development (Cherubini et al., 1991; Ganguly et al., 2001). The precise molecular mechanisms for this shift are still challenging, however, the two cation-chloride cotransporters KCC2 and NKCC1 were identified to play a crucial role in the regulation of Cl^−^ efflux and influx, respectively (Delpire, 2000). During early brain development, expression of NKCC1 is dominant and leads to an intracellular accumulation of Cl^−^ due to the absence of KCC2 in immature neurons (Kanaka et al., 2001; Wang et al., 2002). The expression of KCC2 increases during the first postnatal week, which elevates the efflux of Cl^−^ and causes GABA to become inhibitory (Achilles et al., 2007; Valeeva et al., 2013). The described functional shift of GABA_A_ receptors during postnatal brain development may be involved in the vast receptor density increases we found between P10 and P20 in the olfactory bulb (198%), hippocampus (121%) and cerebellum (907%). Moreover, GABA is the first active neurotransmitter in the immature brain and GABA_A_ receptors are the first to be activated (Sernagor et al., 2010).

NMDA receptors are also present and co-expressed with GABA_A_ on the same postsynaptic side but are initially blocked by Mg^2+^ (Cserép et al., 2012). GABA excitatory actions lead to membrane depolarization and, if strong enough, remove the Mg^2+^ blockage and activate NMDA receptors (Cserép et al., 2012). Therefore, the activation of NMDA receptors opens the voltage-dependent Ca^2+^ channels, leading to an increase in intracellular Ca^2+^ concentration and activation of NMDA receptors in adjacent synapses, resulting in spontaneous synchronous activity (Cserép et al., 2012). This event is crucial for the modulation of DNA synthesis, neuronal proliferation, differentiation, and maturation, as well as for the formation, stabilization, and strengthening of synaptic connections (Cohen et al., 2008; Cherubini et al., 2011; Cserép et al., 2012). We assume that this cascade of events might play a role in the indicated alterations of receptor densities from P10 to P20. Furthermore, the increase occurred during a time period where rats begin to interact with their environment as a result of their eye-opening and hearing onset (Lohmann and Kessels, 2014). Since spontaneous activity depends on GABAergic transmission and can trigger synaptic plasticity, we assume that these development-regulated changes have a high impact on the intense increase in receptor densities found in our study by comparing the postnatal and adult receptor densities.

The significant increase in GABA_A_ receptor densities between P10 and P20 in the olfactory bulb and hippocampus coincides with the timeline of a study concerning the ontogenesis of GABAergic neurons (Coyle and Enna, 1976). It was shown that at birth, GABA binding was approximately 50% of adult levels and that at P8, it was approximately 25% of adult levels, the latter coinciding with our findings in the olfactory bulb and hippocampus (Coyle and Enna, 1976). The authors indicated that the increase after P8 correlated well with an increase in the activity of glutamic acid decarboxylase, a presynaptic marker that is known to catalyze the decarboxylation of glutamate to GABA.

The linear increase in cerebellar GABA_A_ receptor densities is in accordance with the ontogenesis of GABAergic neurons and recognition sites (Coyle and Enna, 1976; Aldinio et al., 1980). Furthermore, the time-course corresponds to the formation of functional GABAergic synapses in the granule cell layer of the cerebellum (Altman, 1972; Shimono et al., 1976). The increase in GABA_A_ receptor densities we found in the olfactory bulb is in accordance with an increase in GABA levels during postnatal brain development (Miranda-Contreras et al., 2000). An increase in both, receptor densities and GABA levels, could be due to neurogenesis of olfactory bulb interneuron populations, in particular the granule cells, which change between postnatal and young adult life (Mair et al., 1982; Bayer, 1983). Future ontogenetic *in situ* or immunohistochemically studies could provide an insight into alterations of receptor subtypes in the individual layers of the olfactory bulb, hippocampus, and cerebellum, but also into other brain regions that undergo developmentally changes such as the thalamic nuclei, the visual, and the auditory cortex.

The early peak of GABA_B_ receptor densities between P0 and P10 is in contrast to the later peak of GABA_A_ receptors in the adult stage, and could be explained by the fact that metabotropic receptors tend to be expressed early in brain development and play a more modulatory role in adult brains (Herlenius and Lagercrantz, 2001). However, the reason is not fully understood yet and would be an interesting aim for future studies. Moreover, GABA_A_ receptors are predominately postsynaptic, whereas GABA_B_ receptors are predominately presynaptic. Therefore, future studies could focus on the differential analysis of alterations in pre- and post-synaptic GABA receptors during development, e.g., *via* electrophysiological methods.

Investigations of the splice variants GABA_B1a_ and GABA_B1b_ revealed that the GABA_B1a_ levels were high at birth, peaked at P5 and then decreased until adulthood, which in part coincides with the decreasing receptor densities in the olfactory bulb from P0 to P30, but does not explain the increasing densities from P30 to P90 (Fritschy et al., 1999). In contrast, GABA_B1b_ levels were low at birth, increased after P5, peaked at P10 and subsequently decreased during maturation, while both types reached adult levels in the third postnatal week (Fritschy et al., 1999). This peak is in accordance with our findings in the hippocampus and striatum, with receptor densities increasing from P0 to P10, when they reached their maximum, and then decreasing but staying at approximately the same levels until P90. Turgeon and Albin (1994) revealed a similar developmental pattern in GABA_B_ binding sites in both ROIs (Turgeon and Albin, 1994). Furthermore, the increase between P0 and P10 corresponds to the time-course and peak of synaptogenesis, which is thought to be regulated by the secretion of brain-derived neurotrophic factor and to follow activation of the tropomyosin receptor kinase pathway (Gaiarsa and Porcher, 2013).

Although their involvement in neuronal development is known, the detailed role of GABA_B_ receptors during ontogenesis is still not adequately understood. It was shown that GABA_B_ levels are highest during the peak of brain development (Turgeon and Albin, 1994), and to a certain extent, we can confirm this with the present study. Nevertheless, the cause of these developmental patterns is still an enigma. Further experiments could help to gain new valuable knowledge, e.g., about the expression pattern of GABA_B_ genes during brain development via *in situ* hybridization.

The present study revealed a comparable pattern of receptor density changes of the glutamatergic AMPA and kainate receptor. In general, their respective densities increased from birth during the first weeks of life and decreased slightly until adulthood, even though not significantly. On the contrary, the GABA receptors investigated revealed differential patterns of density changes, differing depending on the respective receptor type and ROI. The highest differences with respect to percental density change from one age to the next were found for AMPA and GABA_A_ receptors.

## Conclusion

The present study described developmental alterations in glutamatergic and GABAergic receptor densities in the rat brain from P0 to P90. All investigated receptors were present at either P0 or P10, depending on the region of interest, which indicates that each receptor plays a distinct role in postnatal brain development. The increase in glutamatergic receptor densities in the hippocampus, with peaks either at P20 or at P30, implies the possible involvement of these receptors in synaptic plasticity, whereas the decline of NMDA receptor densities in the striatum and hippocampus from P30 to P90 could be caused by synaptic elimination. Taken together, the present data demonstrated regionally specific and time-dependent differential changes of glutamate and GABA receptor densities in the olfactory bulb, striatum, hippocampus, and cerebellum of the rat, thereby contributing to the development of a better understanding of rat brain ontogenesis.

## Data Availability Statement

The datasets generated for this study are available on request to the corresponding author.

## Ethics Statement

All experiments were performed according to the German animal welfare act and were approved by the responsible governmental agency, LANUV NRW (Regional authorities for nature, environment and consumer protection NRW, Germany).

## Author Contributions

SB, JC, MC and KZ designed the concept and experiments. SB performed the experiments and collected the data. NP-G performed statistical analysis. All authors analyzed and discussed the data. SB, JC, NP-G and KA wrote the manuscript.

## Conflict of Interest

The authors declare that the research was conducted in the absence of any commercial or financial relationships that could be construed as a potential conflict of interest.

## Abbreviations

[^3^H]: tritium
AMPA: α-amino-3-hydroxy-5-methyl-4-isoxazolepropionic acid
ANOVA: analysis of variance
GABA: γ-aminobutyric acid
NMDA: N-methyl-D-aspartate
P: postnatal
ROI: region of interest.
